# Comparison of Glycomacropeptide with Phenylalanine Free-Synthetic Amino Acids in Test Meals to PKU Patients: No Significant Differences in Biomarkers, Including Plasma Phe Levels

**DOI:** 10.1155/2018/6352919

**Published:** 2018-01-08

**Authors:** Kirsten K. Ahring, Allan M. Lund, Erik Jensen, Thomas G. Jensen, Karen Brøndum-Nielsen, Michael Pedersen, Allan Bardow, Jens Juul Holst, Jens F. Rehfeld, Lisbeth B. Møller

**Affiliations:** ^1^The PKU Clinic, Kennedy Centre, Centre for Paediatric and Adolescent Medicine, Copenhagen University Hospital, Rigshospitalet, Copenhagen, Denmark; ^2^Department of Clinical Genetics, Applied Human Molecular Genetics, Kennedy Center, Rigshospitalet, Denmark; ^3^Centre for Inherited Metabolic Diseases, Centre for Paediatric and Adolescent Medicine, Copenhagen University Hospital, Rigshospitalet, Copenhagen, Denmark; ^4^Arla Foods Ingredients Group P/S, Viby J, Denmark; ^5^Department of Biomedicine, Aarhus University, Aarhus, Denmark; ^6^Department of Clinical Medicine, Aarhus University Hospital, Aarhus, Denmark; ^7^Department of Odontology, Copenhagen University, Copenhagen, Denmark; ^8^Institute of Clinical Medicine, Copenhagen University Hospital, Rigshospitalet, Copenhagen, Denmark; ^9^Department of Clinical Biochemistry, University of Copenhagen, Rigshospitalet, Copenhagen, Denmark

## Abstract

**Introduction:**

Management of phenylketonuria (PKU) is achieved through low-phenylalanine (Phe) diet, supplemented with low-protein food and mixture of free-synthetic (FS) amino acid (AA). Casein glycomacropeptide (CGMP) is a natural peptide released in whey during cheese-making and does not contain Phe. Lacprodan® CGMP-20 used in this study contained a small amount of Phe due to minor presence of other proteins/peptides.

**Objective:**

The purpose of this study was to compare absorption of CGMP-20 to FSAA with the aim of evaluating short-term effects on plasma AAs as well as biomarkers related to food intake.

**Methods:**

This study included 8 patients, who had four visits and tested four drink mixtures (DM1–4), consisting of CGMP, FSAA, or a combination. Plasma blood samples were collected at baseline, 15, 30, 60, 120, and 240 minutes (min) after the meal. AA profiles and ghrelin were determined 6 times, while surrogate biomarkers were determined at baseline and 240 min. A visual analogue scale (VAS) was used for evaluation of taste and satiety.

**Results:**

The surrogate biomarker concentrations and VAS scores for satiety and taste were nonsignificant between the four DMs, and there were only few significant results for AA profiles (not Phe).

**Conclusion:**

CGMP and FSAA had the overall same nonsignificant short-term effect on biomarkers, including Phe. This combination of FSAA and CGMP is a suitable supplement for PKU patients.

## 1. Introduction

Phenylketonuria (PKU) is an inborn error of metabolism. If left untreated, severe brain damage will occur [1–3]. The primary aim of treatment of those suffering from PKU is to control the blood phenylalanine (Phe) concentration in order to prevent neurological damage [2]. PKU treatment is based on a low-protein (LP) diet in combination with free-synthetic (FS) amino acid (AA) supplements without Phe and enriched with vitamins, minerals, trace elements, and in some products also fat and carbohydrates [1, 4, 5]. Dietary AA supplements are administered to obtain optimal metabolic control by ensuring adequate levels of essential AAs. Diet for life is recommended [6]. Compliance becomes a challenge over time, especially in adolescence, and they are often related to disagreeable taste and the current limitations in available dietary products [7, 8]. In order to achieve better compliance, the formulation of the AA supplement should satisfy the need for better taste and easier management [9]. Therefore, alternatives to conventional treatment are investigated.

Casein glycomacropeptide (CGMP) is a 64-amino acid peptide from cheese whey, which is rich in specific essential AAs and is the only known natural protein free from Phe [10–14]. Hence, CGMP is an alternative source of protein for PKU patients. In this study, we tested the product Lacprodan CGMP-20, a highly purified CGMP product with minimum 95% CGMP and a low level of Phe (0.16 g/100 g AA) and above 78% protein. The residual amount of Phe is due to the presence of minor amounts of other proteins/peptides. However, to ensure the supplement is nutritionally adequate, it requires supplementation of the following AAs to meet the standards of similar PKU supplements: tyrosine (Tyr), tryptophan (Trp), arginine (Arg), histidine (His), leucine (Leu), lysine (Lys), and methionine (Met) [15–17]. Lacprodan CGMP-20 will be referred to as CGMP in this paper.

In this single-blinded, prospective, crossover intervention study, we investigated the utilization and metabolic short-term effect on absorption of pure CGMP-20, FSAA, and a combination of both, all consumed with a standardized meal, by comparing selected relevant surrogate biomarkers. We also evaluated taste and satiety. A second aim was to investigate whether the small amount of Phe in CGMP-20 affected the plasma Phe concentration significantly.

## 2. Materials and Methods

### 2.1. Protocol

The study was approved by the National Committee on Health Research Ethics prior to the study (ID H-3-2014-115), and all participants gave written consent prior to start of the study.

### 2.2. Patient Recruitment

Patients were contacted by mail and invited to participate. They were recruited from the clinical PKU database, including all PKU patients diagnosed and living in Denmark. Patients received compensation for lost wages and travel expenses. Participants in the project were invited to participate if they met the following inclusion criteria: diagnosis of classical PKU confirmed by mutation analysis and a known phe tolerance (12–20 mg/kg) [18–20], age ≥ 15 years at inclusion, had received treatment with a protein-restricted diet since the neonatal period, and were willing and able to visit the PKU clinic four times. The period between visits varied from 24 hours to 1 month, since we estimated this to be sufficient time for a wash-out period [21, 22]. Exclusion criteria were (1) <15 years at inclusion, (2) had not followed the dietary treatment continuously, (3) had a second chronic disease or condition, which potentially could influence the PKU treatment and outcome, (4) treated with BH4, or (5) pregnant, nursing, or planning to become pregnant. Eight patients accepted to participate.

### 2.3. Study Design

The primary purpose of this study was to compare the absorption rate and absorbed amount of peptide-bound AAs (CGMP-20^∗^) (both in its pure form or supplemented with selected FSAAs) with an almost identical mixture of FSAAs with the aim of evaluating the short-term effect on Phe and other AAs as well as biomarkers related to food intake. All patients had four visits in the PKU clinic. The patients consumed a different drink mixture (DM1, DM2, DM3, and DM4) in random order at each visit (blinded to the participants). The order of the DM was randomized by a doctor at the PKU clinic, who was not otherwise involved in the study. CGMP/AA supplements: four different AA sources were tested. DM1: Lacprodan CGMP-20. DM2: FSAA (equivalent AA profile as DM1). DM3: Lacprodan CGMP-20 and synthetic AA. DM4: FSAA (equivalent AA profile as DM3 but without Phe). DM2 had the same AA profile as DM1 consisting of pure CGMP in order to evaluate this; DM3 consisted of CGMP supplemented with FSAA to make it nutritionally adequate and suitable for patients with PKU and had a similar AA profile as DM4 (though this was without the 0.16 g Phe/100 g AA present in CGMP). The crossover study design made it possible to evaluate the effect of the Phe content in GMP. Patients arrived fasting to the clinic in the morning and then subjected to the first venous blood samples (4 ml) at time 0. Subsequently, blood samples were drawn at 15, 30, 60, 120, and 240 minutes (min) after finishing the meal. Taste was evaluated right after consumption. At the end of the visit, all participants were asked to evaluate satiety.

The test meals consisted of a few selected food items (homemade LP bread, butter, and jam) with individually calculated amounts of fat and carbohydrates and only a minimal content of protein and Phe from LP bread. The test meal was consumed in combination with DM, and the individual intake was designed to cover 25% of the daily requirement. In each meal, the total content of protein was equivalent to 25% of 1 g per kg body weight per day (1 g/kg/d). The composition of the meal was calculated after the Nordic Nutrition Recommendations (NNA) and met the criteria for sex, age, and weight: 10–15% from protein, 30–35% from fat, and 50–60% from carbohydrate. After completing the trial, all participants would continue their usual diet and AA supplements. An example of a meal is shown in Table 1, and the content of DM1–4 is given in Table 2.

### 2.4. Blood Samples

All participants had the following blood samples drawn at start (time 0) before consuming the meal/DM and at the end of the study (240 min): glucose, insulin, glucagon-like peptide-1(GLP-1), blood urea nitrogen (BUN), peptide tyrosine-tyrosine (PYY), cholecystokinin (CCK), ghrelin, and AA profiles. Furthermore, ghrelin and AA profiles were (besides at time 0) also measured at 15, 30, 60, 120, and 240 min.

#### 2.4.1. BUN, Glucose, Insulin, Ghrelin, GLP-1, PYY, and CCK

2–4 ml of blood was collected for each biomarker and handled immediately according to protocols [23–28], placed on ice, spinned at 2500 g for 10 min at 4°C, and transferred to specific glasses for further analyses. EDTA glasses for (1) GLP-1 were added 200 *µ*l DPP-IV inhibitor and for (2) ghrelin were added 100 *µ*l Pefabloc by using a 0.5 ml syringe The samples for the total plasma AA profile were frozen at −80°C and sent on dry ice for quantitative analysis of AA using the stable-isotope dilution technique and HPLC-MS/MS [29].

#### 2.5. Visual Analogue Scale (VAS)

This scale (http://www.vastranslator.com) was presented to patients as a horizontal line, ranking from “very hungry” (“0”) to “very satisfied” (“100”) and from “bad taste” (“0”) to “good taste” (“100”) as an application (APP) on iPad. The patients were asked to evaluate the taste of the DM shortly after intake and after 240 min to determine the level of satiety.

#### 2.6. Body Mass Index (BMI) and Fat Percentage

These were measured at visit 1 with a body fat monitor (BF306, Omron Healthcare, USA).

### 2.7. Diet Registration

The patients had filled out a dietary record covering the 24 hours before each visit. They were instructed to eat similar food items every time to ensure that the Phe level would be within the same range the day after. The registrations were calculated with Dankost (http://dankost.dk/english).

### 2.8. Statistical Methods

All calculations were performed using the software SPSS 22 or Microsoft Excel 2010 for windows. Paired and unpaired *t*-tests and one- and two-way ANOVA performed at a significance level of *α* = 0.05 were used. The statistical significance level was at *p* < 0.05.

## 3. Results

### 3.1. Clinical Protocol

Eight patients (seven females, one male), age 15–48 years (mean 33.25 ± SD 11.21), weight 47–85 kg (mean 72.8 + SD 15.9), and height 162–179 cm (mean 170.1 + SD 5.4) were included in the study. Data for each patient are presented in Table 3. Six out of eight (75%) patients completed the study. Patient ID#4 had difficulties eating the meal within 15 min at visit one, which subsequently delayed blood sampling up to 10 min for each blood sample. Patient ID#4 completed day 1 and day 2 as planned, but on the third day, blood sampling was impossible, and for that reason, visit 4 was cancelled and the patient excluded. Patient ID#5 completed visit 1 and visit 2 but was unable to complete day 3 and day 4 due to health problems.

### 3.2. Diet Registration

The 24-hour diet record confirmed similar intake for each patient prior to the four test days.

### 3.3. Test Meals and DM

Intake of protein from DM supplement was 25% of the daily recommendation of 1 g protein/kg/day (mean volume 151.8 g (range 97.9–195.8)), and the test meal provided fat and carbohydrates (Table 4). All patients complied with the intake.

### 3.4. AA Profiles

AA profiles were compared in subgroups (DM1 versus DM2 and DM3 versus DM4), since the respective groups were identical concerning the AA composition. Statistical results were calculated between these subgroups.

The area under the curve (AUC) (adjusted for baseline) for total AA demonstrated insignificant differences between DM1 and DM2 (*p* =0.852) as well as between DM3 and DM4 (*p*=0.06).

We did find significant differences for the following individual AA for AUC: DM1 and DM2: Lys (*p*=0.0287), Asn (*p* =0.0210), and Asp (*p* =0.0047) and DM3 and DM4: citrulline (*p* =0.0162). Results for all the AUC for each AA are presented in Table 5. The highest value for the AUC for the individual AA was Ala, Val, Ile, and Asp (DM1), Pro (DM2), and Leu (DM3).

### 3.5. Plasma Concentrations of AA

The peak plasma concentrations for AAs (given as mean values in percentage of the premeal level) were as follows: DM1: the peak serum concentrations were reached after 30 min for 19 of 21(90%) AAs. Glu reached a peak after 15 min; Tyr only decreased compared to baseline. DM2: four AAs peaked after 30 min, while 15 (71%) AAs peaked after 15 min. Citrulline peaked after 60 min; Tyr decreased compared to time 0. DM3: majority of the AAs peaked after 30 min (67%) except for Asp, Met, and Gln, where the peaks were reached after 15 min. Phe, Glu, citrulline, and Gly all decreased compared to baseline. DM4: fifteen AAs (71%) peaked after 15 min, only five after 30 min, while citrulline peaked after 60 min. All results are displayed in Table 6.

Since most of the AAs peaked after either 15 or 30 min, comparison of the concentrations for each DM after 15 min versus 30 min was performed to test for significant differences between these time points. The following AAs showed significant changes between time 15 and time 30: DM1: Ala (0.0474), Pro (0.0174), Val (0.0299), and Ile (0.0294), DM2: Leu (0.0295), and DM4: Asp (0.0423). There were no significant changes for any AA in DM3. More details are provided in Supplemental Table A.

The paired *t*-test was used for comparison of the concentrations found in DM1 and DM2 and in DM3 and DM4, respectively, at each time point, to test for significant differences between the four DMs. We found no significant differences at time 0. Plasma concentrations (*µ*mol/l) after intake of the DM and test meal for all AAs presented as %. Comparison between DM1+2 and DM3+4 gave the following results: time 15: DM1+2: Tyr: 0.0228, Asp: 0.0136, and citrulline: 0.0378; DM3+4: Pro: 0.0455, Val: 0.0204, and Thr: 0.0239; time 30: DM1+2: Leu: 0.0178, Ile: 0.0021, Asn: 0.0038, and Asp: 0.0362; DM3+4: Ser: 0.0031, Pro: 0.0486, Thr: 0.0027, His: 0.0108, Tyr: 0.0412, and citrulline: 0.0409; time 60: DM1+2: Asp: 0.0060 and citrulline: 0.0100; DM3+4: Pro: 0.0003, Val: 0.0088, Thr: 0.0270, and Asp: 0.0155; time 120: DM3+4: citrulline:0.0364; and time 240: DM1+2: Asp: 0.0288. There were no significant differences at time 0.

Compared to baseline values, ghrelin values demonstrated a significant decrease at 30, 60, and 120 min for DM1, at 30 min for DM2, at 60 and 120 min for DM3, and at 30, 60, and 120 min for DM4, respectively. Final levels decreased 7% for DM1, increased 10% for DM2 and 5% for DM3, and remained unchanged in DM4, all compared to baseline.  Table 7 displays results (% compared to baseline) and the *t*-test for all levels and values (mean and SD) in Supplemental Table B.

**Figure 1:**
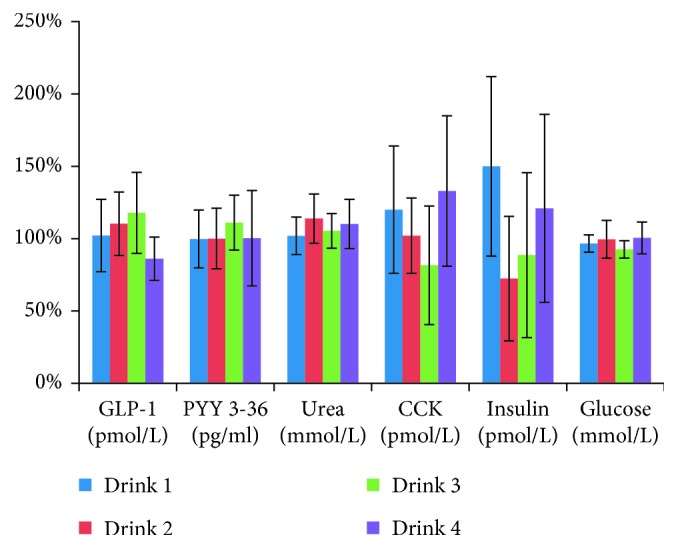
Results (mean +/− SD) for the following biomarkers: glucose, insulin, GLP-1, PYY, BUN, and CCK. None of them demonstrated significant changes from baseline (premeal) to the end of the study period (240 min after meal and DM).

None of the biomarkers GLP-1, PYY, BUN, CCK, insulin, and glucose showed significant changes from baseline (premeal) to the end of the study period (240 min after meal and DM). Results for biomarkers are presented in Figure 1 (% compared to baseline) and values (mean +/− SD) in Supplemental Table C.

### 3.6. VAS Score

The question “how satisfied are you?” obtained the following nonsignificant results: DM1 (mean 36, SD 18), DM2 (mean 41, SD 16), DM3 (mean 28, SD 27), and DM4 (mean 35, SD 30). The results for the other question “how does the DM taste?” were as follows: DM1 (mean 46, SD 31), DM2 (mean 44, SD 22), DM3 (mean 36, SD 28), and DM4 (mean 26, SD 22). All comparisons (DM1 and DM2, DM3 and DM4, but also DM3 compared to DM1 and DM2, resp.) were statistically insignificant.

## 4. Discussion

This study evaluated the metabolic short-term effect of CGMP compared to an almost identical combination of FSAA by repeated measurements. One of the most important findings was that the residual content of Phe in DM3 did not affect the plasma level significantly compared to DM4 at any time which support data from Ney et al. [11].

Over the last 8 years, CGMP has been investigated in mice studies and a few human studies to evaluate safety, acceptability, and efficacy of CGMP as a nutritional supplement for treatment of PKU [11–13, 15, 30, 31]. The present study supports the conclusions of these studies.

The slower absorption of most of the AA in DM1 and DM3, which contained CGMP, compared to DM2 and DM4, which contained only FSAA, could be explained by the fact that CGMP delay the absorption in the gut. The fact that Tyr increased in DM3 and DM4, but only decreased without peak in DM1 and DM2, is assumed to be caused by the low content in DM1 and DM2. Tyr and Trp both peaked at 30 min for DM3 compared to 15 min for DM4, suggesting that the content of Tyr and Trp in the CGMP mixtures were metabolized less rapidly than the FSAA. The Phe/Tyr ratio decreased over time with 30% in both DM3 and DM4, while it increased (caused by the low-Tyr concentration in DM1 and DM2) with 50% for DM1 and 70% for DM2. The Phe/Tyr ratio is an important measure because a high ratio can have a long-term negative effect on executive functions [32].

The AUC for “total AA” was not associated with absorption rate. We also calculated the AUC for each AA and compared with peak values to determine complete absorption and absorption rate, and we did find significant differences for Lys, Asn, and Asp for DM1 and DM2 and for citrulline for DM3 and DM4. None of the LNAA (extra-added in DM3 and matched in DM4) was significantly different.

Ala, Pro, Val, and Ile demonstrated a significant increase from 15 to 30 min for DM1, while only Leu in DM2 and Asp in DM4 decreased significantly, which indicate a better absorption of pure CGMP. The fact that none of the AA in DM3 changed significantly could be the influence of the extra-added AA (LNAA and Lys). In contrast, we did see significant differences between several AAs by comparison between DM1 and DM2 and between DM3 and DM4 at each time point. Especially His, Tyr, and Trp are noteworthy for DM3 and DM4, since they are FSAAs added to the pure CGMP. Also, Thr, Ile, and Val are of certain interest, since the natural content of these three AAs in CGMP is high [33]. The high content of these AAs and addition of extra-LNAA to the CGMP offer an additional positive effect, since LNAA competes for transport across the blood-brain barrier (BBB) via the L-type amino acid transporter (LAT1) [34, 35]. High Phe in plasma diminishes uptake of Tyr and Trp into the brain, and this results in reduced formation of neurotransmitters [36]. This imbalance is possibly the major cause of disturbed brain development in PKU patients [37]. Each LNAA has individual affinity relation to LAT1 [38]. Several studies have shown the positive blocking effect of LNAA which reduces Phe entering the brain [39–41]. Matalon et al. [17] demonstrated a decrease in Phe in the blood up to 50% using LNAA tablets and emphasized that LNAA in a specific mixture inhibits Phe uptake already in the gut [16, 42]. Administrations of Val, Ile, and Leu have proved to reduce Phe concentrations in the cerebrospinal fluid of humans [43].

All biomarkers remained unchanged by comparing time 0 and 240 min, and there were no significant changes in plasma Phe despite the residual amount of Phe in CGMP in line with findings from Ney et al. [11, 13]. This study demonstrated that AA in CGMP is absorbed as efficient as an identical mixture of FSAA.

All the DMs demonstrated a decreasing effect on ghrelin after the meal. Ghrelin values showed significant decreases at 30, 60, and 120 min for DM1, at 30 min for DM2, at 60 and 120 min for DM3, and at 30, 60, and 120 min for DM4. Low-ghrelin concentrations are associated with a feeling of satiety [44, 45]. By only evaluating DM1 and DM2, it could indicate that satiety is reached faster for CGMP. However, VAS scores for satiety did not show any significant difference between DM1 and DM2, nor between DM3 and DM4.

BUN was nonsignificantly lower for DM1 and DM3 compared to DM2 and DM4, which potentially could suggest a more efficient utilization of GMP compared to AA, as found by van Calcar et al. [13]. Similar findings have been reported by Ney et al. [46]. However, our present short-term study was not able to support these findings.

GLP-1 promotes insulin secretion and reduces appetite and reached the highest (nonsignificant) level with DM3 after 240 min (118%) which may indicate that satiety was better obtained with DM3 compared with DM1, DM2, and DM4. This finding concurs with previous studies, showing that GMP promotes satiety [14]. PYY also reduces appetite and reached the highest value for DM3 (111%). CCK is a peptide hormone in the gastrointestinal system responsible for stimulating the digestion of fat and protein. The wide variation from a decrease of 18% in DM3 to an increase of 33% in DM4 was unexpected, since CCK is expected to rise after a mixed meal [23].

A limitation of this study was the small number of patients. However, it was important for the study design to select as homogeneous a test population as possible (only early-treated classical PKU confirmed by mutation analysis), resulting in exclusion of a number of patients.

Three patients had a BMI over 30 and were defined as obese, one slightly obese, three had a normal BMI, and one had a BMI below normal. The patients were receiving dosage after their actual weight, which means that the patients that were categorized with normal BMI received less DM per kg lean body mass.

Although this study demonstrated nonsignificant changes for almost all biochemical markers, it is important to notice that this study only replaced a single meal and a long-term effect could be different. Based on the current results, we consider CGMP to be a safe alternative to FSAA but should be supplemented with additional FSAA to make it nutritionally adequate and potential also with other nutrients as fat, carbohydrates, vitamins, and minerals to create an easy-to-use supplement for patients with PKU. If CGMP products substitute FSAA completely, it is necessary to carefully monitor if the small content of Phe will have an impact on the blood level in the long term. If Phe levels increase, restrictions of the LP diet must be implemented to balance and control the Phe intake and blood level. Further studies are needed to evaluate the long-term impact and efficacy of CGMP in the management of PKU.

## 5. Conclusion

Dietary management of PKU should be lifelong, and good compliance is crucial for a good outcome. CGMP did not change any of the biomarkers significantly compared to free-synthetic AA when consumed with a test meal in PKU patients. The residual amount of Phe in CGMP did not affect the plasma Phe level significantly. Based on these data, we consider CGMP to be a suitable alternative as supplement for PKU treatment. However, further research is needed to determine the long-term effects and safety of CGMP. This study demonstrates that CGMP has the same short-term effect as FSAA.

## Figures and Tables

**Table 1:** Example of a test breakfast meal including DM (calculated individually for each participant).

DM (CGMP-20, AA, or CGMP+AA)
2 slices (80 g) of LP bread
Butter (20 g)
Jam (20 g)

**Table 2:** Content of pure CGMP (g AA/100 g p) and DM1–4 (pr. 1000 g mixture) (CGMP and free-synthetic AA). DM1: 100% CGMP: 158.46 g = the content of each AA displayed below. DM2: 100% FSAA (the total amount of AA is shown below). DM3: 119.04 g from CGMP (+AA = the additional amount of AA from FSAA). DM4: 100% FSAA.

CGMP-20	AA	DM1: Lacprodan CGMP-20: 158.46 g^∗^	DM2: FSAA	DM3: Lacprodan CGMP-20: 119.04 g^∗^ + FSAA	DM4: FSAA
g AA/100 g protein	Total amount of AA (g)				
6.4	Ala	8.57	8.40	6.44	6.31
0.3	Arg	0.44	0.43	4.85^∗^	4.85
9.2	Asp	12.08	11.83	9.07	8.89
0.08	Cys	0.15	0.15	0.11	0.11
21.1	Glu	26.53	25.99	19.93	19.52
1.2	Gly	1.53	1.50	1.15	1.13
0.2	His	0.16	0.16	3.0^∗^	3.04
11.5	Ile	14.50	14.21	10.89	10.67
2.5	Leu	3.12	3.06	12.11^∗^	12.06
6.4	Lys	8.46	8.29	7.37^∗^	7.24
2	Met	2.92	2.85	3.62^∗^	3.57
0.2	Phe	0.20	0.20	0.15	0
12.6	Pro	16.45	16.11	12.36	12.10
8.5	Ser	10.46	10.25	7.86	7.70
18.1	Thr	23.62	23.14	17.74	17.38
0.04	Trp	0.00	—	2.44^∗^	2.44
0.06	Tyr	0.05	0.05	10.81^∗^	10.81
9.5	Val	11.76	11.52	8.83	8.65
—	Citric acid powder	—	—	14.40	1.40
—	Citric acid solution (50% w/w in water)	11.20	—	—	—
—	Tropical twist flavour (IFF SC401962)	1.75	1.75	1.75	1.80
—	Sucrose	73.00	73.00	80.00	80.00
—	Water	755.59	787.12	751.95	780.17
109.88	Total	1000.00	1000.00	1000.00	1000.00
—	DM	1	2	3	4
81	Protein equivalent (g/100 g)	12.00	12.00	12.00	12.00
—	Carbohydrate (g/100 g)	7.46	7.30	8.12	8.00
—	Fat (g/100 g)	0.03	0	0.02	0
—	Energy (kcal/100 g)	78	77	81	80

^∗^Part of the AA content comes from CGMP and part comes from additional FSAA: Arg: 0.33 (CGMP) + 4.52 (AA) = 4.85, His 0.12 (CGMP) + 2.92 (AA) = 3.04, Leu: 2.35 (CGMP) + 9.76 (AA) = 12.11, Lys: 6.36 (CGMP) + 1.01 (AA) = 7.37, Met: 2.19 (CGMP) + 1.43 (AA) = 3.62, Trp: 0.00 (CGMP) + 2.44 (AA) = 2.44, Tyr: 0.04 (CGMP) + 10.77 (AA) = 10.81.

**Table 3:** Patient data, all with classic PKU: age, mutations, height, weight, BMI, and usual AA supplement.

Patient ID#	Age	Mutation 1	Mutation 2	Phenotype	Height (cm)	Weight (kg)	BMI	Usual AA product
1	48	c.1315+1G>A^1^	c.1315+1G>A^1^	Classic PKU	169	85	30	PreKUnil tablets
2	27	c.1315+1G>A^1^	c.1222C>T^2^	Classic PKU	174	62	20	XPhe energy
3	18	c.842C>T^3^	c.1315+1G>A^1^	Classic PKU	171	75	26	Avonil powder
4	16	c.1222C>T^2^	c.1315+1G>A^1^	Classic PKU	171	47	16	Avonil tablets
5	38	c.1315+1G>A^1^	c.1222C>T^2^	Classic PKU	179	79	25	PreKUnil tablets
6	46	c.814G>T^4^	c.1222C>T^2^	Classic PKU	173	94	31	PreKUnil tablets
7	34	c.473G>A^5^	c.1315+1G>A^1^	Classic PKU	162	53	20	PreKUnil tablets
8	39	c.1222C>T^2^	c.1222C>T^2^	Classic PKU	162	87.5	33	Avonil tablets

^1^(IVS12+1G>A); ^2^p.R408W; ^3^p.E280K; ^4^p.G272X; ^5^p.R158Q.

**Table 4:** Intake of DM (water + CGMP mixture powder = total volume) and standard meal in grams (g) and energy (kilojoule (kJ) and energy % (E %)).

Patient ID	1	2	3	4	5	6	7	8	Mean	SD
Powder (g)	41	30	36	21	38	45	27	44	35	8
Water (g)	136	99	120	77	126	151	83	139	116	25
LP bread (g)	70	90	170	100	82	70	96	80	95	30
Butter (g)	23	23	35	26	21	21	23	19	24	5
Marmalade (g)	40	40	40	42	40	40	40	40	40	1
Energy (kJ)	2324	2330	3445	2405	2324	2327	2316	2322	2474	368
Protein (g)	22	16	20	13	20	24	14	23	19	4
Protein E (%)	16	12	10	9	15	18	10	17	13	3
Fat (g)	20	20	32	23	19	18	21	18	21	4
Fat E (%)	32	33	34	35	30	29	33	28	32	2
Carbohydrate (g)	70	75	111	78	74	71	76	74	79	12
Carbohydrate E (%)	52	56	56	57	55	53	57	56	55	2

**Table 5:** Results for the AUC (*µ*mol/l over time) for all AAs (adjusted for baseline (time 0)). Significant differences for the following AAs for the area under the curve (AUC) minus baseline: DM1 and 2: Lys (*p* =0.0287), Asn (*p* =0.0201), and Asp (*p* =0.0046) and DM3 and 4: citrulline (*p* =0.0162).

DM	1		2		3		4	
	Mean	SD	Mean	SD	Mean	SD	Mean	SD
Gly	−1145	12734	11361	20714	−16590	15386	11709	9483
Ala	38797	10277	29471	5292	8397	3693	20653	5239
Ser	8021	2312	6262	680	−1264	2222	2180	1230
Pro	28232	3802	33492	7623	9353	2444	15950	2204
Val	46664	5818	33878	7511	14370	5722	24624	7244
Thr	38627	7843	35865	3144	13558	1338	20698	2662
Leu	−3102	499	−2979	786	6899	1798	5206	1564
Ile	26638	2253	21551	1691	9666	1474	8429	1146
Lys	16623	3356	5005	3255	3932	2076	7342	2542
Asn	3036	688	910	409	1838	978	1129	652
Met	1976	580	1848	230	842	429	3026	1961
His	−419	768	−1133	677	−1036	1151	1003	697
Phe	−6717	11135	−2778	6468	−24356	10869	−8944	9170
Tyr	−3376	592	−4000	649	6672	1012	6019	1622
Glu	123	399	−305	541	−2302	1004	−988	782
Gln	34247	7716	39614	10878	−4902	4414	9870	7528
Asp	1399	413	−254	239	−682	490	−469	217
Trp	−1364	403	−1078	412	804	849	994	484
Orn	1167	818	733	621	799	724	1288	304
Arg	−505	1292	−100	1136	−1121	1310	1047	941
Cit	−1115	634	790	647	−2193	549	305	652

**Table 6:** Plasma concentrations (*µ*mol/l) after intake of the DM and test meal for all AAs presented as % compared to time 0 (=1). Comparison between DM1+2 and DM3+4 gave the following results: time 15: DM1+2: Tyr: 0.0228, Asp: 0.0136, citrulline: 0.0378; DM3+4: Pro: 0.0455, Val: 0.0204, Thr: 0.0239; time 30: DM1+2: Leu: 0.0178, Ile: 0.0021, Asn: 0.0038, Asp: 0.0362; DM3+4: Ser: 0.0031, Pro: 0.0486, Thr: 0.0027, His: 0.0108, Tyr: 0.0412, citrulline: 0.0409; time 60: DM1+2: Asp: 0.0060, citrulline: 0.0100; DM3+4: Pro: 0.0003, Val: 0.0088, Thr: 0.0270, Asp: 0.0155; time 120: DM3+4: citrulline: 0.0364; time 240: DM1+2: Asp: 0.0288. There were no significant differences at time 0.

Time	15		30		60		120		240	
	Mean	SD	Mean	SD	Mean	SD	Mean	SD	Mean	SD
DM1										
Gly	0.961	0.339	1.062	0.616	0.944	0.500	1.011	0.355	0.963	0.717
Ala	1.656	0.435	2.340	0.653	1.940	0.462	1.510	0.419	1.233	0.328
Ser	1.669	0.434	2.189	0.615	1.535	0.434	1.024	0.172	1.194	0.440
Pro	2.904	0.886	3.886	1.117	2.819	1.256	1.782	0.303	1.523	0.374
Val	1.977	0.510	2.509	0.698	2.255	0.422	1.712	0.257	1.583	0.310
Thr	2.594	0.544	3.885	0.867	2.855	0.813	2.013	0.229	1.832	0.829
Leu	1.365	0.152	1.425	0.155	0.995	0.094	0.582	0.162	0.828	0.143
Ile	5.815	1.849	7.033	1.190	5.198	0.841	3.426	0.464	2.603	0.517
Lys	2.195	0.794	2.258	0.500	1.828	0.690	1.468	0.653	1.263	0.734
Asn	1.666	0.251	2.003	0.399	1.412	0.313	1.012	0.091	1.218	0.414
Met	2.255	0.714	2.275	0.684	1.690	0.522	1.165	0.235	1.071	0.387
His	1.000	0.116	1.153	0.245	1.034	0.231	0.842	0.118	1.060	0.339
Phe	0.942	0.130	1.012	0.189	0.990	0.199	0.935	0.131	0.976	0.271
Tyr	0.816	0.143	0.863	0.245	0.765	0.170	0.599	0.134	0.647	0.136
Glu	1.302	0.307	1.243	0.149	1.061	0.180	0.915	0.127	0.961	0.173
Gln	1.278	0.204	1.309	0.296	1.291	0.277	1.245	0.208	1.242	0.402
Asp	1.783	0.495	2.225	0.760	1.592	0.327	1.076	0.245	1.104	0.316
Trp	0.980	0.105	1.116	0.188	0.906	0.155	0.725	0.145	0.808	0.256
Orn	1.288	0.340	1.520	0.501	1.287	0.366	0.989	0.175	1.006	0.325
Arg	1.041	0.135	1.220	0.272	1.024	0.198	0.876	0.193	0.963	0.242
Cit	0.889	0.251	1.075	0.316	0.809	0.228	0.744	0.244	0.939	0.467
DM2										
Gly	1.238	0.926	1.197	0.745	1.081	0.938	1.227	0.796	0.979	0.654
Ala	1.603	0.354	1.664	0.483	1.620	0.447	1.312	0.335	1.163	0.379
Ser	1.653	0.297	1.697	0.546	1.457	0.204	1.102	0.213	1.083	0.169
Pro	3.177	0.867	3.052	1.017	2.846	1.185	2.291	1.110	1.617	0.435
Val	1.647	0.579	1.856	0.730	1.719	0.397	1.544	0.560	1.420	0.497
Thr	2.555	0.512	3.182	0.977	2.808	0.691	2.254	0.735	1.957	0.447
Leu	1.389	0.255	1.225	0.196	0.983	0.094	0.635	0.129	0.810	0.160
Ile	4.892	2.668	4.640	2.055	4.192	1.679	3.112	1.420	2.572	0.857
Lys	1.714	0.725	1.420	0.530	1.280	0.341	1.046	0.218	1.023	0.438
Asn	1.518	0.172	1.314	0.359	1.155	0.124	0.954	0.197	1.046	0.270
Met	1.992	0.542	1.830	0.641	1.570	0.328	1.220	0.173	1.085	0.184
His	1.114	0.208	1.045	0.308	0.993	0.175	0.823	0.178	0.944	0.171
Phe	1.055	0.227	0.952	0.195	0.956	0.109	0.964	0.159	1.036	0.130
Tyr	0.951	0.231	0.812	0.187	0.692	0.132	0.586	0.145	0.606	0.125
Glu	1.130	0.182	1.087	0.177	1.016	0.165	0.897	0.222	0.964	0.202
Gln	1.537	0.427	1.508	0.350	1.478	0.531	1.199	0.393	1.271	0.390
Asp	1.200	0.426	1.132	0.266	0.880	0.155	0.814	0.230	1.005	0.190
Trp	1.075	0.304	0.996	0.361	0.871	0.263	0.768	0.171	0.884	0.171
Orn	1.227	0.290	1.081	0.287	1.067	0.211	1.084	0.302	1.079	0.275
Arg	1.207	0.286	1.111	0.395	1.084	0.215	0.927	0.215	0.931	0.258
Cit	1.118	0.525	1.136	0.404	1.211	0.349	1.121	0.359	1.030	0.359

DM3										
Gly	0.809	0.355	0.913	0.337	0.822	0.621	0.772	0.318	0.804	0.679
Ala	1.206	0.304	1.435	0.302	1.171	0.192	1.085	0.277	0.987	0.141
Ser	1.210	0.211	1.248	0.262	0.941	0.194	0.862	0.292	0.900	0.200
Pro	1.777	0.313	1.838	0.349	1.471	0.234	1.278	0.415	1.126	0.242
Val	1.459	0.274	1.715	0.330	1.228	0.117	1.314	0.572	1.078	0.143
Thr	1.625	0.368	1.851	0.471	1.635	0.217	1.438	0.463	1.325	0.293
Leu	2.282	0.435	2.328	0.442	1.658	0.366	1.091	0.617	0.887	0.141
Ile	3.524	1.089	3.675	0.890	2.498	0.721	1.736	1.234	1.380	0.386
Lys	1.632	0.560	1.662	0.712	1.389	0.599	0.943	0.117	0.874	0.189
Asn	1.630	0.329	1.534	0.429	1.353	0.335	1.034	0.430	0.980	0.202
Met	1.826	0.370	1.754	0.507	1.255	0.116	1.070	0.374	0.865	0.180
His	1.049	0.153	1.153	0.240	0.915	0.137	0.900	0.264	0.895	0.129
Phe	0.889	0.111	0.911	0.104	0.843	0.100	0.879	0.140	0.910	0.119
Tyr	1.645	0.244	1.985	0.713	1.723	0.437	1.667	0.295	1.305	0.248
Glu	0.950	0.144	0.951	0.152	0.801	0.189	0.756	0.221	0.774	0.111
Gln	1.170	0.104	1.061	0.223	1.036	0.120	0.911	0.148	0.920	0.093
Asp	0.953	0.291	1.111	0.138	0.998	0.158	0.755	0.221	0.724	0.170
Trp	1.244	0.272	1.455	0.302	1.165	0.382	1.071	0.444	0.950	0.383
Orn	1.259	0.247	1.374	0.230	1.147	0.222	1.064	0.335	0.977	0.195
Arg	1.216	0.226	1.236	0.331	0.982	0.138	0.869	0.213	0.835	0.163
Cit	0.815	0.158	0.802	0.173	0.649	0.163	0.642	0.141	0.842	0.211
DM4										
Gly	1.179	0.781	1.304	0.630	0.920	0.445	1.230	0.433	1.183	0.690
Ala	1.546	0.395	1.552	0.278	1.463	0.406	1.304	0.157	1.003	0.378
Ser	1.521	0.336	1.419	0.228	1.196	0.382	0.978	0.120	0.961	0.347
Pro	2.499	1.154	2.357	0.774	2.003	0.704	1.509	0.287	1.135	0.429
Val	1.861	0.438	1.865	0.450	1.528	0.297	1.511	0.913	1.263	0.491
Thr	2.067	1.025	2.389	0.795	2.069	0.687	1.654	0.224	1.380	0.525
Leu	2.237	0.667	1.981	0.433	1.579	0.482	0.995	0.277	0.829	0.279
Ile	3.681	1.813	3.072	1.203	2.667	1.251	1.481	0.582	1.132	0.457
Lys	1.671	0.782	1.385	0.292	1.429	0.704	1.184	0.389	1.179	0.589
Asn	1.588	0.591	1.502	0.439	1.291	0.342	1.008	0.191	0.851	0.231
Met	2.527	1.706	2.337	1.887	1.956	2.094	1.503	1.681	1.035	0.763
His	1.237	0.231	1.261	0.135	1.140	0.270	1.004	0.153	0.981	0.342
Phe	1.054	0.126	0.981	0.109	0.947	0.141	0.942	0.108	0.921	0.293
Tyr	1.548	0.594	1.524	0.559	1.606	0.723	1.667	0.568	1.347	0.525
Glu	1.123	0.205	1.013	0.191	0.950	0.221	0.823	0.168	0.893	0.407
Gln	1.236	0.246	1.150	0.160	1.120	0.211	1.107	0.196	0.911	0.319
Asp	1.121	0.261	0.964	0.149	0.854	0.234	0.819	0.177	0.860	0.343
Trp	1.505	0.436	1.403	0.272	1.252	0.345	1.031	0.147	1.010	0.355
Orn	1.470	0.405	1.376	0.200	1.337	0.302	1.105	0.153	0.992	0.314
Arg	1.477	0.315	1.344	0.238	1.138	0.218	0.967	0.116	0.940	0.314
Cit	1.132	0.285	1.105	0.216	1.201	0.603	0.989	0.231	0.971	0.413

**Table 7:** Ghrelin levels over time, presented as % relative to start value (time 0) + SD and *p* value.

	DM1			DM2			DM3			DM4		
Time	Relative (%)	SD (%)	*p* value	Relative (%)	SD (%)	*p* value	Relative (%)	SD (%)	*p* value	Relative (%)	SD (%)	*p* value
0	100	—	—	100	—	—	100	—	—	100	—	—
15	95	12	0.266	88	11	0.056	90	13	0.171	75	22	0.050
30	82	5	0.006^∗^	83	15	0.035^∗^	78	19	0.055	80	10	0.015^∗^
60	81	13	0.030^∗^	84	16	0.068	80	12	0.013^∗^	75	11	0.006^∗^
120	81	10	0.020^∗^	84	15	0.088	75	14	0.011^∗^	74	13	0.010^∗^
240	93	18	0.454	110	20	0.180	105	25	0.473	100	34	0.986

^∗^Significant.

## Abbreviations

CGMP: Casein glycomacropeptide, CGMP-20 (product name Lacprodan CGMP-20)
PKU: Phenylketonuria
AA: Amino acid(s)
FSAA: Free-synthetic AA
BBB: Blood-brain barrier
BUN: Blood urea nitrogen
GLP-1: Glucagon-like peptide-1
CCK: Cholecystokinin
PYY: Peptide tyrosine-tyrosine
DM: Drink mix
Ala: Alanine
Arg: Arginine
Asn: Asparagine
Asp: Aspartate
Cys: Cysteine
Glu: Glutamine
Gly: Glycine
His: Histidine
Ile: Isoleucine
Leu: Leucine
Lys: Lysine
Met: Methionine
Phe: Phenylalanine
Pro: Proline
Ser: Serine
Thr: Threonine
Trp: Tryptophan
Tyr: Tyrosine
Val: Valine
PAH: Phenylalanine hydroxylase
VAS: Visual analogue score
LP: Low protein
min: Minutes
SD: Standard deviation
kJ: Kilojoule.

## Supplementary Materials

Supplementary Materials
*Table A*. Results for all AA at time 0, 15, 30, 60, 120, and 240 (Mean and SD). *Table B*. Ghrelin results for time 0, 15, 30, 60, 120, and 240 (Mean and SD). *Table C*. Results for glucose, insulin, GLP-1, PYY, BUN, and CCK (Mean ± SD). None of them demonstrated significant changes from baseline (pre-meal) to end of study period (240 min after meal and DM).
