# Supplementary material for: Comparison of Glycomacropeptide with Phenylalanine Free-Synthetic Amino Acids in Test Meals to PKU Patients: No Significant Differences in Biomarkers, Including Plasma Phe Levels
Source: J Nutr Metab. 2018 Jan 8;2018:6352919. doi: 10.1155/2018/6352919 (PMC5817308; doi:10.1155/2018/6352919)
Supplement: Supplementary Materials — Table A. Results for all AA at time 0, 15, 30, 60, 120, and 240 (Mean and SD). Table B. Ghrelin results for time 0, 15, 30, 60, 120, and 240 (Mean and SD). Table C. Results for glucose, insulin, GLP-1, PYY, BUN, and CCK (Mean ± SD). None of them demonstrated significant changes from baseline (pre-meal) to end of study period (240 min after meal and DM). [file 6352919.f1.pdf]

Supplementary

Table A: Results for all AA at time 0, 15, 30, 60, 120 and 240 (Mean and SD)

Time (min)	DM1		DM2		DM3		DM4	
Glycine	Mean	SD	Mean	SD	Mean	SD	Mean	SD
0	393	119	369	115	367	147	312	87
15	378	142	456	180	297	93	368	198
30	417	136	441	222	336	103	407	183
60	371	171	398	169	302	94	287	129
120	397	139	452	186	284	76	383	145
240	378	135	361	199	296	79	369	161
Alanine	Mean	SD	Mean	SD	Mean	SD	Mean	SD
0	273	55	328	83	306	104	299	95
15	452	169	526	118	369	137	462	117
30	638	314	546	173	439	220	464	157
60	529	172	532	107	358	138	438	160

120	412	189	431	94	332	62	390	130
240	337	86	382	72	302	68	300	119
Serine	Mean	SD	Mean	SD	Mean	SD	Mean	SD
0	107	22	104	25	103	24	100	26
15	179	51	172	25	125	28	153	51
30	235	110	177	67	129	38	142	44
60	165	45	152	39	97	12	120	40
120	110	22	115	34	89	16	98	24
240	128	46	113	22	93	11	96	32
Proline	Mean	SD	Mean	SD	Mean	SD	Mean	SD
0	102	21	106	28	111	29	105	31
15	296	109	338	68	197	49	262	40
30	396	110	324	91	204	75	247	58
60	287	93	302	121	163	28	210	42
120	182	37	243	125	142	34	158	32
240	155	48	172	38	125	26	119	48
Valine	Mean	SD	Mean	SD	Mean	SD	Mean	SD
0	229	46	250	100	217	46	209	48
15	452	132	412	85	317	81	389	57
30	574	172	464	161	373	124	390	94
60	516	111	430	82	267	44	319	79
120	391	95	386	84	285	111	316	163
240	362	90	355	42	234	35	264	105
Threonine	Mean	SD	Mean	SD	Mean	SD	Mean	SD
0	123	65	111	33	117	47	117	57

15	320	183	283	79	190	58	242	35
30	479	333	352	194	216	113	279	109
60	352	154	311	83	191	70	242	109
120	248	128	249	50	168	37	193	77
240	226	108	217	47	155	42	161	86

Leucine	Mean	SD	Mean	SD	Mean	SD	Mean	SD
0	93	16	87	23	86	21	93	22
15	128	27	121	25	196	57	208	53
30	133	26	107	33	200	75	184	51
60	93	22	86	25	143	29	147	40
120	54	18	55	16	94	28	93	22
240	77	18	71	16	76	17	77	28

Isoleucine	Mean	SD	Mean	SD	Mean	SD	Mean	SD
0	37	8	38	15	38	11	40	14
15	218	86	186	32	134	43	146	47
30	263	61	176	61	140	58	122	38
60	195	48	159	28	95	30	106	25
120	128	27	118	28	66	22	59	17
240	97	26	98	24	52	9	45	19

Lysine	Mean	SD	Mean	SD	Mean	SD	Mean	SD
0	118	38	131	33	135	13	112	30
15	258	144	224	62	220	68	188	52
30	265	108	186	31	224	90	156	37
60	215	64	168	57	187	61	161	55
120	173	67	137	27	127	21	133	18
240	148	45	134	38	118	32	132	48

Asparagine	Mean	SD	Mean	SD	Mean	SD	Mean	SD
0	47	8	45	10	47	9	45	10
15	77	21	68	13	76	15	72	16
30	93	29	59	18	72	27	68	16
60	66	15	51	10	63	11	58	17
120	47	9	43	8	48	8	46	10
240	57	17	47	7	46	5	38	10

Tyrosine	Mean	SD	Mean	SD	Mean	SD	Mean	SD
0	46	9	50	10	47	5	47	12
15	37	6	48	9	77	11	73	9
30	40	7	41	12	93	25	72	22
60	35	9	35	8	81	13	75	26
120	27	10	29	8	78	19	78	17
240	30	7	30	5	61	15	63	19

Phenylalanine	Mean	SD	Mean	SD	Mean	SD	Mean	SD
0	809	431	827	268	901	410	747	334
15	762	368	872	343	801	375	787	334
30	819	393	786	248	821	377	733	350
60	801	441	790	280	760	363	707	334
120	757	386	797	316	792	406	703	332
240	790	368	856	289	819	393	688	326

glutamic acid	Mean	SD	Mean	SD	Mean	SD	Mean	SD
0	45	6	45	14	50	16	44	18
15	59	13	50	12	47	12	49	19
30	56	7	48	11	47	11	45	16
60	48	14	45	11	40	7	42	15

120	41	10	40	13	38	7	36	10
240	43	9	43	10	38	9	39	15

glutamine	Mean	SD	Mean	SD	Mean	SD	Mean	SD
0	563	107	527	143	678	85	594	98
15	719	199	810	72	793	92	735	90
30	737	131	795	158	719	130	684	154
60	727	87	779	121	702	147	666	163
120	701	124	632	106	618	103	658	144
240	699	131	670	221	624	34	541	152

Aspartic acid	Mean	SD	Mean	SD	Mean	SD	Mean	SD
0	18	6	16	5	18	9	15	6
15	32	10	19	5	18	3	17	4
30	39	19	18	6	21	10	15	5
60	28	9	14	5	18	7	13	5
120	19	8	13	3	14	3	13	4
240	20	7	16	5	13	5	13	6

Tryptophan	Mean	SD	Mean	SD	Mean	SD	Mean	SD
0	36	9	33	10	33	11	32	7
15	35	9	36	10	40	9	48	6
30	40	14	33	12	47	18	44	14
60	32	7	29	9	38	9	40	14
120	26	9	25	5	35	5	33	8
240	29	11	29	9	31	4	32	12

Ornithine	Mean	SD	Mean	SD	Mean	SD	Mean	SD
0	41	8	36	10	34	6	33	7

15	53	12	44	10	43	9	48	11
30	63	22	39	9	47	11	45	10
60	53	13	38	8	39	7	44	13
120	41	8	39	12	36	8	36	7
240	41	6	39	8	33	5	32	10

Arginine	Mean	SD	Mean	SD	Mean	SD	Mean	SD
0	41	8	36	10	34	6	33	7
15	53	12	44	10	43	9	48	11
30	63	22	39	9	47	11	45	10
60	53	13	38	8	39	7	44	13
120	41	8	39	12	36	8	36	7
240	41	6	39	8	33	5	32	10

Citrulline	Mean	SD	Mean	SD	Mean	SD	Mean	SD
0	32	9	29	9	34	5	29	7
15	28	6	33	11	28	7	32	4
30	34	13	33	6	27	7	32	10
60	26	6	35	11	22	5	34	14
120	24	5	33	12	22	5	28	9
240	30	7	30	5	29	7	28	9

Methionine	Mean	SD	Mean	SD	Mean	SD	Mean	SD
0	20	3	22	4	22	5	21	6
15	46	17	44	10	39	12	53	23
30	46	17	40	15	38	18	49	26
60	34	8	34	8	27	7	41	29
120	24	6	27	4	23	5	32	23
240	22	7	24	4	19	4	22	11

Histidine	Mean	SD	Mean	SD	Mean	SD	Mean	SD
0	74	15	67	17	16	17	67	18
15	74	20	75	14	19	20	83	18
30	85	34	70	26	28	29	85	23
60	77	22	67	24	14	15	77	30
120	62	19	55	9	12	13	67	16
240	79	22	63	14	11	12	66	27

Table B: Ghrelin results for time 0, 15, 30, 60, 120 and 240 (Mean and SD)

	DM 1		DM 2		1&2	DM 3		DM 4		3&4	all
time	Mean	SD	Mean	SD	p-value	Mean	SD	Mean	SD	p-value	p-value
0	1402	665	1265	564	0,71	1435	582	1297	575	0,69	0,95
15	1327	633	1110	432	0,5	1297	619	971	396	0,29	0,6
30	1154	540	1044	446	0,71	1114	618	1038	383	0,8	0,97
60	1140	488	1059	464	0,77	1148	512	970	404	0,51	0,89
120	1135	496	1062	399	0,78	1079	522	961	393	0,66	0,92
240	1301	464	1392	558	0,76	1505	566	1299	456	0,5	0,89

Table C: Results for glucose, insulin, GLP-1, PYY, BUN and CCK (Mean +/- SD). None of them demonstrated significant changes from baseline (pre-meal) to end of study period (240 min after meal and DM)

drink	1			2			3			4		
	baseline	end	P-value	baseline	end	P-value	baseline	end	P-value	baseline	end	P-value
Glucose	5,42 +/- 0,30	5,23 +/- 0,34	0,334	5,27 +/- 0,42	5,24 +/- 0,58	0,935	5,4 +/- 0,27	4,99 +/- 0,36	0,071	5,28 +/- 0,30	5,3 +/- 0,40	0,906
insulin	62,46 +/- 26,45	70,43 +/- 15,65	0,563	83,22 +/- 41,08	73,54 +/- 47,13	0,726	57 +/- 36,40	71,96 +/- 21,80	0,484	69,16 +/- 37,78	82,21 +/- 39,31	0,537
GLP-1	20,43 +/- 4,31	20,86 +/- 4,64	0,871	19,57 +/- 3,58	21,57 +/- 4,66	0,420	18 +/- 3,00	21,2 +/- 2,71	0,131	22,5 +/- 3,43	19,38 +/- 2,39	0,068
PYY	51,43 +/- 12,10	51,29 +/- 7,52	0,981	48,71 +/- 12,26	48,71 +/- 9,75	1,000	39,33 +/- 6,67	43,67 +/- 8,24	0,382	51,5 +/- 11,03	51,63 +/- 14,02	0,985
BUN	3.18 +/- 0.41	3,61 +/- 0,81	0,260	3.25 +/- 0.83	3,37 +/- 0,78	0,800	3,41 +/- 0,99	3,37 +/- 0,36	0,937	3,28 +/- 0,74	3,27	0,991
CCK	1,33 +/- 0,45	1,6 +/- 0,81	0,523	1,41 +/- 0,47	1,44 +/- 0,39	0,910	1,53 +/- 1,21	1,25 +/- 0,25	0,620	1,16 +/- 0,37	1,54 +/- 0,79	0,297
